# An Environment-Wide Association Study (EWAS) on Type 2 Diabetes Mellitus

**DOI:** 10.1371/journal.pone.0010746

**Published:** 2010-05-20

**Authors:** Chirag J. Patel, Jayanta Bhattacharya, Atul J. Butte

**Affiliations:** 1 Department of Pediatrics and Medicine, Stanford University School of Medicine, Stanford, California, United States of America; 2 Stanford Center for Biomedical Informatics Research, Stanford University School of Medicine, Stanford, California, United States of America; 3 Lucile Packard Children's Hospital, Palo Alto, California, United States of America; 4 Center For Primary Care and Outcomes Research, Stanford University School of Medicine, Stanford, California, United States of America; East Carolina University, United States of America

## Abstract

**Background:**

Type 2 Diabetes (T2D) and other chronic diseases are caused by a complex combination of many genetic and environmental factors. Few methods are available to comprehensively associate specific physical environmental factors with disease. We conducted a pilot Environmental-Wide Association Study (EWAS), in which epidemiological data are comprehensively and systematically interpreted in a manner analogous to a Genome Wide Association Study (GWAS).

**Methods and Findings:**

We performed multiple cross-sectional analyses associating 266 unique environmental factors with clinical status for T2D defined by fasting blood sugar (FBG) concentration ≥126 mg/dL. We utilized available Centers for Disease Control (CDC) National Health and Nutrition Examination Survey (NHANES) cohorts from years 1999 to 2006. Within cohort sample numbers ranged from 503 to 3,318. Logistic regression models were adjusted for age, sex, body mass index (BMI), ethnicity, and an estimate of socioeconomic status (SES). As in GWAS, multiple comparisons were controlled and significant findings were validated with other cohorts. We discovered significant associations for the pesticide-derivative heptachlor epoxide (adjusted OR in three combined cohorts of 1.7 for a 1 SD change in exposure amount; p<0.001), and the vitamin γ-tocopherol (adjusted OR 1.5; p<0.001). Higher concentrations of polychlorinated biphenyls (PCBs) such as PCB170 (adjusted OR 2.2; p<0.001) were also found. Protective factors associated with T2D included β-carotenes (adjusted OR 0.6; p<0.001).

**Conclusions and Significance:**

Despite difficulty in ascertaining causality, the potential for novel factors of large effect associated with T2D justify the use of EWAS to create hypotheses regarding the broad contribution of the environment to disease. Even in this study based on prior collected epidemiological measures, environmental factors can be found with effect sizes comparable to the best loci yet found by GWAS.

## Introduction

It is becoming clear that most non-communicable diseases are a result of a complex combination of genetic processes and the environment [1]. Despite the contribution of both genetics and environment to disease, many recent studies have emphasized the genetic components. For example, the Genome-wide Association Study (GWAS) is a low-cost and popular framework used by researchers to evaluate genetic factors that correlate with disease status on a genome-wide scale [2]. As of this writing, 370 publications using this method have been cataloged, with 16 just for Type 2 Diabetes Mellitus (T2D) [3]. Multiple loci markers have been found through these studies that heightens risk for T2D when present [4]. While GWAS has enabled the generation of new hypotheses regarding the relation of genetics to T2D, the genetic markers found have poor penetrance [5], [6]. Further, these genetic markers do not explain a significant portion of T2D in context of other factors [7], [8].

Perhaps the lack of impact of GWAS comes from not comprehensively considering environmental factors in disease. T2D provides an specific example: while genetics play a large role [9]–[11], specific environmental factors are also emerging as risk factors for the disease [12]. It is clear that we need to measure and assess both types of factors to better understand complex disease [1].

The current paradigm to search for the effects of multiple environmental chemicals utilizes molecular tools and model systems [13], [14]; however, there is a gap between these data and human disease. Epidemiological searches for environmental factors associated with disease have been hampered by the lack of a “chip” or standard bioassays that can broadly survey these factors. We propose borrowing the GWAS methodology to create a model Environmental-Wide Association Study (EWAS), to search for environmental factors associated with disease on a broad scale. This type of study is made possible by the use of cross-sectional epidemiological data, the National Health and Nutrition Examination Survey (NHANES), a nationally representative, biannual health survey conducted by the Centers For Disease Control and Prevention (CDC) [15]. Participants are queried regarding their health status and an extensive battery of clinical and laboratory tests are performed on a subset of these individuals. Specific environmental attributes are assayed, such as chemical toxicants, pollutants, allergens, bacterial/viral organisms, and nutrients.

The EWAS consists of two methodological steps that have analogs in a GWAS. First, we consider a panel of 266 unique environmental assays, or environmental “loci”, measured across cases of diabetics and controls, yielding several environmental factors with significantly high association with T2D while controlling for multiple hypotheses. Second, we validate the associations by taking advantage of data from other cohorts in NHANES. With EWAS, we are able to hypothesize about new associations with T2D and reconfirm others. The results from EWAS can better inform about environmental factors that need to be measured in genetic studies to begin to provide us insight in regards to disease etiology.

## Methods

### Ethics Statement

The NHANES is a publicly available dataset made available by the CDC and National Centers for Health Statistics and all participants have provided written consent.

We associate 266 unique environmental factors with T2D status from the NHANES. We downloaded the all of the available NHANES data for 1999–2000, 2001–2002, 2003–2004, and 2005–2006 cohorts and collated corresponding variables across them. For example, if a variable with symbol *LBXVIE* from 1999–2000 described “A-Tocopherol ug/dL” and variable with symbol *LBXATC* from 2001–2002 also described “a-tocopherol ug/dL”, we harmonized onto the single symbol for both, *LBXATC*.

**Figure 1:**
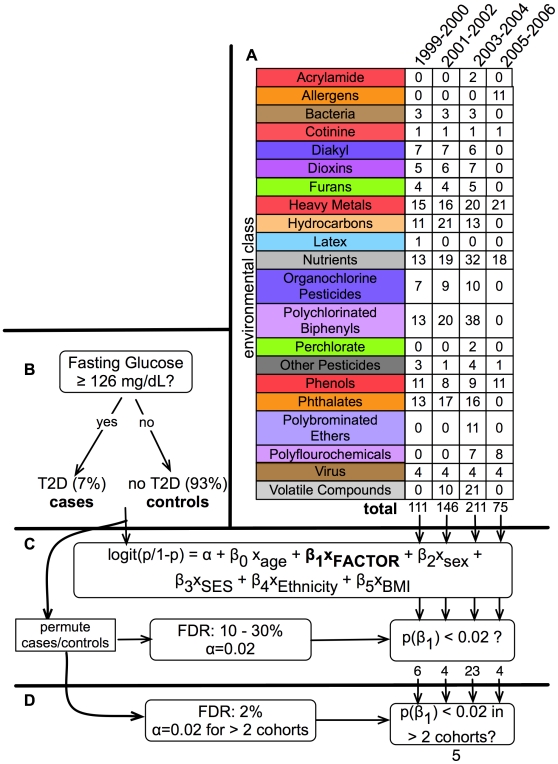
Summary of EWAS Environment Factors and Analytic Method. A.) Summary of the 21 factor classes and the number of factors within them for each NHANES cohort. B.) Individuals were stratified into T2D status (“cases” and “controls”) through the ADA FBS threshold for diabetes diagnosis. 6–7% of the unweighted observations in all cohorts had T2D under this rule. C.) Each of these 75 to 211 factors was tested for association with T2D status with a logistic regression model (coefficient labeled “FACTOR”) adjusted for age, sex, BMI, ethnicity, and SES. Statistical significance (α = 0.02) was determined by controlling the FDR between 10 to 30%. Between 4 and 23 factors were found to be significant using this threshold α = 0.02. D.) “Multi-stage” validation. For factors that were deemed significant in C, we deemed a factor validated if the factor was significant to the α = 0.02 level in one or more of the other cohorts. We found 5 factors to be validated (FDR of 2%).

Figure 1 presents a schematic representation of our analysis methodology. We analyzed all environmental factors from the NHANES that were a direct measurement of an environmental attribute, such as the amount of pesticide or heavy metal present in urine or blood. We did not consider internal biological system laboratory measures such as red blood cell count, triglyceride level, cholesterol level, or other physiological measures. By using direct and quantitative measures of factors, we potentially avoid issues of self-report bias.

There was a total of 543 factors in our EWAS, but not all factors were present in all cohorts: 111 factors measured in the 1999–2000 cohort, 146 from 2001–2002, 211 from 2003–2004, and 75 from 2005–2006. This comprised of 266 unique environmental factors in total, with 157 factors measured in more than one cohort. Using NHANES categorization, we binned factors into 21 “class” groupings in order to discern patterns among related groups of factors, analogous to chromosomal units in GWAS (Figure 1A). Different environmental factors were measured in varying numbers of participants, ranging from 507 to 3318 individuals over the different environmental factors.

We omitted from our EWAS 73 factors that varied little across individuals in our sample. Specifically, we omitted those that had a majority (>90%) of the observations below a detection limit threshold as defined by in the NHANES codebook. We also removed factors that targeted a subset of the population, such as the test for *Trichomonas vaginalis*, an infectious pathogen found primarily in women.

T2D cases were individuals who had a fasting blood glucose (FBG) level greater or equal to 126 mg/dL, as advised by the American Diabetes Association (ADA) [16] (Figure 1B). We chose specificity and accuracy of diagnosis over sensitivity, as we acknowledge this definition ignores those who were previously diagnosed as diabetic, but now keep their blood glucose under tight control; in fact, a larger proportion of NHANES respondents described themselves as diabetics or were taking medications often used to treat diabetes than were classified by FBG levels. Neither FBG nor the self-reported diabetes status distinguishes between Type 1 Diabetes (T1D) and T2D; as T2D has a prevalence rate more than 40 times higher than T1D, we assumed all our cases have T2D. This claim is further justified given the average age of the participants considered were between 41 and 42 years of age for all cohorts.

We used survey-weighted logistic regression to associate each of the 543 environmental attributes with diabetes status while adjusting for age, sex, body mass index (BMI), ethnicity, and an estimate for SES (Figure 1C). We acknowledge that estimating SES is difficult; nevertheless, we used the tertile of poverty index, equivalent to the participant's household income divided by the time-adjusted poverty threshold, as the estimate for SES. We used *R* with the *survey* module to conduct all survey-weighted analyses, and replicated our results with the STATA program [17]–[19].

Exposures were captured either as continuous or a categorical variable. Most chemical exposure data arising from mass spectrometry or absorption measurements occurred within a very small range and had a right skew; thus, we log transformed these variables. Further, we applied a z-score transformation (adjusting each observation to the mean and scaling by the standard deviation) in order to compare odds ratios from the many regressions. Similarly, for categorical variables, we made the definition of the referent consistent, defining them to be the “negative” results of the test.

We calculated the false discovery rate (FDR), the estimated proportion of false discoveries made versus the number of real discoveries made at a given significance level, to control for type I error due to multiple hypotheses testing in associating the factors to disease status [20]. To estimate the number of false discoveries, we created a “null distribution” of regression test statistics by shuffling the diabetes status labels 1000 times and recomputing the regressions. The FDR was then estimated to be the ratio of the proportion of results that were called significant at a given level α in the null distribution and the proportion of results called significant from our real tests. To choose factors significantly associated with T2D in the first single-cohort phase, we used a significance level (α = 0.02), which corresponded to a FDR of 10% across three out of four cohorts (1999–2000, 2003–2004, and 2005–2006) and 30% for the 2001–2002 cohort.

To improve our power, we used the four independent cohorts to validate significant findings (Figure 1D). We considered a significant factor as “validated” if it was found to be significant (α = 0.02) in more than one cohort, at the expense of having to drop those factors not measured in a second cohort. We then assessed the FDR of the multi-cohort validation. We first estimated the number of false positives by counting the number of factors found significant at a level α in two or more cohorts from the permuted datasets. We then estimated the FDR by computing the ratio between the number of false positives and the number of validated factors. This value was 2% with α equal to 0.02.

We fit a final logistic regression model with data combined from multiple NHANES cohorts utilizing all measurements for a specific environmental factor, attaining an overall odds ratio. The covariates of the final model were age, sex, BMI, ethnicity, SES, and cohort. We computed new sample weights for the combined datasets by taking the average of the original sample weights as described by the NHANES analytic guidelines [21].

We conducted 3 secondary analytic tests for the validity and sensitivity of our final estimates. We first attempted to check for reverse causality, or association of exposure due to T2D diagnosis. Our second test attempted to take into account the lipophilic characteristics of the environmental factors found. Our last test attempted to take into account recent food and supplement consumption as a potential bias for exposure measures. For adequate sample size and ease of comparison to the final fit model, we utilized all available data combining multiple NHANES cohorts as the sample to conduct these three tests, described below.

To attempt to account for reverse causality, we recomputed our models omitting those individuals who had been diagnosed with diabetes, defined here as those answering yes answers submitted on a NHANES health questionnaire (“Doctor told you have diabetes?”). We then refit our final models with individuals only showing biochemical evidence of T2D without actual diagnosis.

Our second test attempted to account for the lipophilic chemical characteristics of our significant factors. Many of the environmental factors measured in NHANES absorb readily in fatty tissue; presence of fatty tissue is also associated with T2D and a potential confounder. Thus, we recomputed the models taking into account total triglycerides and cholesterol measured in blood specimen of participants.

In our third test, we attempted to compare dietary and supplement consumption of cases or controls gathered from 24- and 48-hour recall and supplement use questionnaires reasoning that recent intake may confound exposure-disease association. The NHANES data contains amount of food components consumed based on the dietary recall available for all participants examined above. Specifically, amounts of food components are computed from the questionnaire using the United States Department of Agriculture (USDA) Food and Nutrient Database. Some of the vitamin and nutrient components included vitamin A, vitamin B-6, vitamin B-12, vitamin C, vitamin E, vitamin K, carotenes, lycopene, thiamin, riboflavin, niacin, folate, calcium, iron, magnesium, phosphorus, potassium, sodium, iron, zinc, copper, and selenium. Other components included macronutrients, such as protein, carbohydrates, fat, fiber, and cholesterol. The total amount of food components considered numbered 51 to 63 for the different cohorts. Further, the 2003–2004 and 2005–2006 cohorts contained both 24- and 48-hour recall data. Supplement use included count of consumption of vitamins, minerals, botanicals, and/or their mixture of them over the past month prior to the survey. To check for possible confounding by recent consumption, we added each food and supplement variable to the logistic regression models specified above and re-evaluated significance and effect size of the validated environmental factors. We coded food component content as the logarithm (base 10) of the amount entered. We coded supplement use as an integer count value. We acknowledge the potential of bias with the use of questionnaire data and a pre-determined database of food items but assumed it was a reliable proxy of consumption and behavioral data in lieu of other information.

## Results

Using GWAS as inspiration, we systematically and comprehensively assessed the association of 266 unique environmental factors measured in the NHANES with T2D. Further, we validated these associations by observing the significance of factors in other NHANES cohorts.

### Population characteristics

File S1 describes the baseline and demographic characteristics of people who were considered as diabetics for our data. Across the cohorts, the total non-weighted and weighted numbers of those who were diabetic compared to non-diabetic were similar. However, we did see significant differences with demographic factors such as sex, age, and socioeconomic status between cases and controls. T2D occurred in higher age groups in all cohorts (p<0.001, two-sided t-test). There were significantly more male participants than females in all cohorts (p<0.001, 0.02, 0.03, χ^2^ test) except for 2005–2006. Furthermore, there was a significant association between lowest SES (first tertile of poverty index) and T2D (p = 0.006, 0.03, 0.04, logistic regression) in for the 1999–2000, 2001–2002, and 2005–2006 cohorts respectively. While we did not see a univariate association between ethnicity and T2D as diagnosed by FBG, we did confirm previously reported associations of ethnicity with T2D when stratifying by age and sex [22]. As expected, BMI was significantly associated with T2D status (p<0.001, t-test) for all cohorts. Given these differences between the cases and controls, we adjusted our logistic regression models described below accordingly.

### Environment Associations with T2D

**Figure 2:**
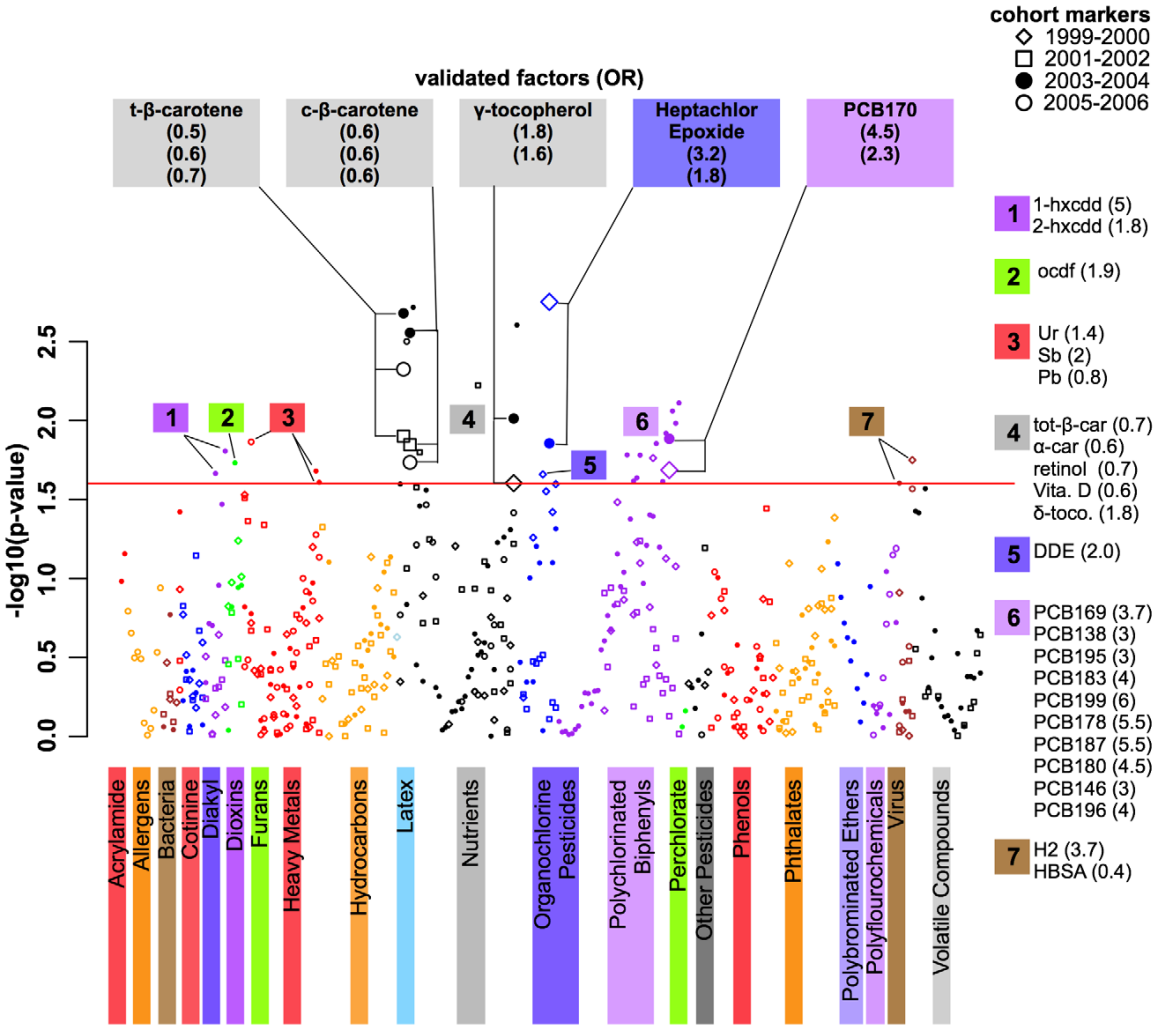
“Manhattan plot” style graphic showing the environment-wide association with T2D. Y-axis indicates −log10(p-value) of the adjusted logistic regression coefficient for each of the environmental factors. Colors represent different environmental classes as represented in Figure 1A. Within each environmental class, factors are arranged left to right in order from lowest to highest odds ratio (OR). Plot symbols represent different cohorts: 1999–2000 (diamonds), 2001–2002 (square), filled dot (2003–2004), circle (2005–2006). Red horizontal line is −log10(α) = 1.8 (α = 0.02). Validated factors significant in 2 or more NHANES cohorts are in bold face (α = 0.02 in two or more cohorts, FDR of 2%) with larger plot points. Other significant factors (α = 0.02) are annotated with numeric label corresponding to the environmental factor class color key on the right. Figure abbreviations: Validated factors: *t-β-carotene*: trans β-carotene; *c-β-carotene*: cis β-carotene; *PCB170*: 2,2′,3,3′,4,4′,5-Heptachlorobiphenyl. Group 1 (dioxins): *1-hxcdd*: 1,2,3,6,7,8-Hexachlorodibenzo-p-dioxin; *2-hxcdd*: 1,2,3,7,8,9-Hexachlorodibenzo-p-dioxin. Group 2 (furans): *OCDF*: 1,2,3,4,6,7,8,9-Octachlorodibenzofuran. Group 3 (heavy metals): *Ur*: uranium; *Sb*: antimony; *Pb*: Lead. Group 4 (nutrients): *tot-β-car*: total β-carotene; *α-car*: alpha-carotene; *retnl*: retinol; *Vita. D*: vitamin D; *δ-t*: delta-tocopherol. Group 5 (organochlorine pestcides): *DDE*: dichlorodiphenyltrichloroethylene. Group 6 (PCB): *PCB169*: 3,3′,4,4′,5,5′-hexachlorobiphenyl; *PCB138*: 2,2′,3,4,4′,4′,5-Hexachlorobiphenyl; *PCB195*: 2,2′,3,3′,4,4′,5,6-Octachlorobiphenyl; *PCB183*: 2,2′,3,4,4′,5′,6-Heptachlorobiphenyl; *PCB199*: 2,2′,3,3′,4,5,5′,6′-Octachlorobiphenyl; *PCB178*: 2,2′,3,3′,5,5′,6-Heptachlorobiphenyl; *PCB187*: 2,2′,3,4′,5,5′,6-Heptachlorobiphenyl; *PCB180*: 2,2′,3,4,4′,5,5′-Heptachlorobiphenyl; *PCB146*: 2,2′,3,4′,5,5′-Hexachlorobiphenyl; *PCB196*: 2,2′,3,4,4′,5,5′,6-Octachlorobiphenyl. Group 7 (bacteria): *H2*: Herpes Simplex 2; *HSBA*: Hepatitis B Surface Antibody.

Figure 2 shows the distribution of p-values of association for each environmental factor and class, adjusted for sex, age, BMI, ethnicity, and the estimate for SES, plotted in a “Manhattan plot” analogous to the association results from a GWAS study. The 37 significant or notable factors are annotated in the figure. Specific categories show association with T2D, such as organochlorine pesticides, nutrients/vitamins, polychlorinated biphenyls, and dioxins (Figure 2 and Table S1), having between 10 to 30% of the factors in the class with p-values less than 0.02. Many positive (low p-values) and negative (high p-values) associations replicated well among the different cohorts.

Table 1 shows those factors that were validated as being significant in two or more of the independent cohorts (multi-cohort validation FDR of 2%). Predicted probabilities of having T2D were computed for a prototype participant, a 45 year old white male with BMI of 27 (middle of the range for non-diabetics in the NHANES sample) and from the middle SES, at high and low exposure levels. For combined cohorts, the predicted probability applies to a prototype participant from the 2005–2006 cohort. We also computed the overall estimate by combining NHANES cohort data in a final model additionally adjusted for cohort; the predicted probabilities for these models were computed for a prototype participant as defined above. We defined low exposure as having a log transformed exposure level one standard deviation lower than the transformed mean, and high exposure as having a log transformed exposure level one standard deviation higher than the transformed mean. For example, a 45-year-old male from the 1999–2000 cohort with high levels (0.09 ng/g) of heptachlor epoxide has a 6% likelihood of being in our diabetes subset. Figures S1, S2, S3, S4, and S5 show the distributions of raw exposure levels per diabetes status.

**Table 1:** Highly statistically significant environmental factors associated with T2D found in more than one NHANES cohort.

Environmental class	Environment Factor	Cohort	N† T2D, No T2D	P	OR (95% CI)	Factor Level (Lo-Hi)	Predicted Probability (Lo-Hi)
Nutrients	cis-β-carotene	2001–2002	211, 2852	0.01	0.6 (0.5–0.8)	0.4–1.4 ug/dL	0.12–0.05
		2003–2004	207, 2698	0.002	0.63 (0.5–0.7)	0.4–1.9	0.13–0.06
		2005–2006	186, 2425	0.02	0.6 (0.5–0.8)	0.4–1.6	0.15–0.06
		2001–2006*	604, 7975	<0.001	0.6 (0.5–0.7)	0.4–1.7	0.15–0.06
	trans-β-carotene	2001–2002	211, 2854	0.01	0.6 (0.5–0.8)	5.1–27.2 ug/dL	0.13–0.05
		2003–2004	207, 2698	0.002	0.7 (0.6–0.8)	4.8–24.7	0.13–0.06
		2005–2006	203, 2701	0.004	0.6 (0.4–0.7)	4.8–29.0	0.16–0.06
		2001–2006 *	621, 8253	<0.001	0.6 (0.5–0.7)	4.9–27.0	0.15–0.06
	γ-tocopherol	1999–2000	146, 2091	0.02	1.8 (1.3–2.4)	107–360 ug/dL	0.03–0.09
		2003–2004	207, 2698	0.01	1.6 (1.3–2.0)	103–356	0.06–0.13
		1999–2006*	767, 10307	<0.001	1.5 (1.3–1.7)	107–352	0.06–0.13
Organochlorine Pesticides	Heptachlor Epoxide	1999–2000	46, 635	0.002	3.2 (2.4–4.4)	0.02–0.09 ng/g	0.01–0.06
		2003–2004	67, 809	0.01	1.9 (1.3–2.6)	0.01–0.07	0.02–0.07
		1999–2004*	178, 2367	<0.001	1.7 (1.3–2.1)	0.02–0.08	0.03–0.07
Polychlorinated Biphenyls	PCB170	1999–2000	45, 716	0.02	2.3 (1.5–3.6)	0.03–0.12 ng/g	0.01–0.06
		2003–2004	53, 773	0.01	4.5 (2.1–9.9)	0.01–0.12	0.03–0.42
		1999–2004*	165, 2426	<0.001	2.2 (1.6–3.2)	0.02–0.13	0.04–0.15

Odds ratio for each exposure, adjusted for BMI, age, sex, ethnicity, and SES is calculated for a change in the log exposure level by one standard deviation, along with the 95% confidence interval. Factor level is the amount of exposure defined by the low (1 SD lower than the average logged exposure level) and high range (1 SD higher than the average logged exposure level). The predicted probability range is an estimate for a 45-year-old white male with BMI of 27 kg/m^2^ from the middle SES to develop the disease in the low to high range of exposure.

* denotes analysis using combined NHANES cohorts; models adjusted for age, sex, ethnicity, BMI, SES, and cohort; predicted probabilities for combined cohorts applies to an individual from the 2005–2006 cohort.

†denotes unweighted number.

### Nutrients and Vitamins: Carotenes and γ-tocopherol

Several vitamins were found to have levels inversely associated with T2D. The first type included an antioxidant in the isoforms of β-carotene (final adjusted odds ratio (OR) of 0.6; 95% confidence interval (CI) 0.5–0.7; p<0.001). For the prototypical participant, high levels of trans or cis β-carotene equated to a 9% improvement in risk (15 vs. 6%) for T2D status. We were able to confirm the inverse association of β-carotenes seen in multiple epidemiological studies in Saudi Arabia [23], among older people [24], among Swedish men [25], and in an earlier NHANES III cohort (pre-1999) [26], as well as another small study that showed an inverse response between fasting glucose level and β-carotene [27]. However, in a prospective case-control study β-carotene was not significantly inversely associated with T2D [28]. Because T2D is associated with reduced anti-oxidant defense, anti-oxidants, such as carotenes, have been occasionally recommended as a therapy [29]. However, the evidence of mitigation of T2D with these vitamins as therapies has been negligible in clinical trials, including women who are high risk of cardiovascular disease [30] or male smokers [31].

We discovered a vitamin that increased risk for T2D. Surprisingly, γ-tocopherol, a form of vitamin E, was highly significantly and positively associated with T2D (final adjusted OR 1.5; 95% CI 1.3–1.7; p<0.001) in two cohorts (adjusted OR of 1.8 and 1.6; p = 0.02 and 0.01 for 1999–2000 and 2001–2002 cohorts) and nearly significant in the two others (adjusted OR of 1.3 and 1.6; p = 0.06 and 0.04 for 2001–2002 and 2005–2006 cohorts). For the prototypical participant, low levels of the γ-tocopherol equated to a 7% improvement in risk (13% vs. 6%). To our knowledge, this is a novel association between γ-tocopherol and T2D.

### Persistent Pollutants: Polychlorinated Biphenyls and Organochlorine Pesticides

We found organochlorinated pesticides and polychlorinated biphenyls (PCBs), both related pollutant factors, to be a highly positively associated with T2D. Among the PCBs, we specifically discovered PCB170 (2,2′,3,3′,4,4′,5-Heptachlorobiphenyl; final adjusted OR of 2.2; 95% CI 1.6–3.2; p<0.001). The effect sizes in the individual cohorts for PCB170 were large (adjusted OR 2.3 and 4.5; p = 0.02 and 0.01 for 1999–2000 and 2003–2004 cohorts). The models predicted up to 15% T2D risk for the prototype participant, more than double the risk of those with low concentrations of PCB170. The association between the class of PCBs with T2D has been well described within Native American [32], Japanese [33], Swedish [34], and Taiwanese [35] cohorts.

Heptachlor epoxide, an oxidation product of the organochlorine pesticide heptachlor, was among the most highly associated factor (final adjusted OR 1.7; 95% CI 1.3–2.1; p<0.001) in our EWAS. The effect sizes in the individual cohorts were also large (adjusted OR 3.2 and 1.9; p = 0.002 and 0.01 for 1999–2000 and 2003–2004 cohorts). The predicted probability for the prototypical participant with high levels of the pollutant was 7%, more than 2 times greater than those who had lower levels of this pollutant.

### Secondary analysis to test validity of the final estimates

We then attempted to test the validity of our final estimates by conducting 3 additional analytic tests. In the first test, we attempted to consider the possibility of “reverse causality” or differential exposure status due to T2D diagnosis. Second, we attempted to assess the effect of potential confounding bias due to the lipophilic characteristics on our final environmental factor effect estimates. Third, we attempted to assess the effect of recent nutrient and supplement consumption on our final effect estimates.

To consider T2D diagnosis as a modulator of exposure, we removed all individuals who answered yes when questioned about a past history of diabetes in the NHANES health questionnaire (“Doctor told you have diabetes?”). We then recomputed the effect of exposure, adjusted for age, sex, SES, ethnicity, BMI, and cohort using the remaining individuals who showed biochemical evidence of T2D, but not carrying a diagnosis of T2D (Table S2). For all validated factors significant in more than 2 cohorts above (Table 1), the estimates remained stable and statistically significant. The effect size for Heptachlor Epoxide was marginally smaller with an adjusted OR of 1.6 (95% CI 1.1–2.1; p = 0.008). The adjusted OR for PCB170 was also marginally smaller, 2.1 (95% CI 1.2–3.9; p = 0.02). The effect of γ-tocopherol was larger, with an adjusted OR of 1.8 (95% CI 1.3–2.2; p<0.001) and there was no change to effect sizes of the carotenes (adjusted OR 0.6; 95% CI 0.5–0.7; p<0.001). We concluded that there was not enough evidence to support the phenomenon of reverse causality based on the effect sizes estimated for those who were at risk for T2D.

We next attempted to account for potential confounding bias of lipid levels. To assess the degree of possible confounding we refit the logistic regression adjusting for the logarithm (base 10) of total triglyceride and cholesterol levels in addition to age, sex, BMI, SES, ethnicity, and cohort (Table S3). We did not observe a great change in effect sizes estimates for the environmental factors after this further adjustment for total triglycerides and cholesterol. The odds ratio after adjusting for lipid levels for carotenes was 14% higher, 0.7 (95% CI 0.6–0.8; p<0.001) compared to 0.6 (Table 1). Similarly, the odds ratio for γ-tocopherol was attenuated by 7%, 1.4 (95% CI 1.2–1.6; p<0.001) compared to 1.5 (Table 1). For the pesticide factor, the odds ratio was smaller by 6%, 1.6 (95% CI 1.3–2.0; p<0.001) versus 1.7 (Table 1). Lastly, for PCB factor, we observed a 3% higher odds ratio of 2.3 (95% CI 1.4–3.7, p = 0.002) versus 2.2 (Table 1). Consistent with this secondary analysis, we observed a similar degree of effect size differences when using the “Lipid Adjusted” NHANES environmental factors, which are only provided for few of the pollutant factors (not shown). We concluded that the effect sizes of the environmental factors were affected by lipid levels, but not substantially biased by them.

We then searched for differences in food and supplement consumption patterns between diabetics and non-diabetics for all 4 cohorts close to the time of survey derived from dietary recall and supplement use questionnaires. In comparing dietary nutrients, we did not observe a difference for any dietary nutrient except one between cases and controls. This exception included a lower total carbohydrate intake for diabetics versus controls, confirming that many diabetics may have known about their disease; specifically, the adjusted OR was 0.7 (95% CI 0.6–0.8; p = 0.001) for a 10% increase in total carbohydrate consumption, adjusted for sex, age, ethnicity, SES, and cohort. We also observed an inverse association between any supplement use and T2D, with an adjusted OR of 0.6 (95% CI 0.5–0.8, p<0.001), also consistent with our expectation of increased health awareness for those with T2D. However, we specifically could find no difference in consumption of carotenes or tocopherol (p = 0.85 and 0.2 respectively) between cases and controls, two of the validated nutrient factors found in our EWAS (Table 1).

Having observed some difference in consumption behavior between cases and controls, we then attempted to assess the influence of recalled dietary consumption on the environmental associations by recomputing the logistic regression models in presence of dietary and supplement use variables. Adding the new dietary or supplementary vitamin consumption variables did not attenuate the odds ratios (maximum change of 1–2%), nor did they lessen the strength of the associations for all of the 5 validated environmental factors described in Table 1. Thus, we did not have evidence to support that recent consumption influenced the factor-disease effect sizes for the validated factors found in our EWAS.

We took a further step in assessing the strength of the environmental associations, adjusting for total triglycerides and cholesterol, any supplement use, and food intake simultaneously (Table S4). Specifically, the odds ratio for a SD increase in γ-tocopherol levels was 1.3 (95% CI 1.1–1.5; p = 0.004) when adjusting for logarithm base 10 of triglycerides, cholesterol, total vitamin E consumption, beta carotene consumption, total carbohydrate consumption, and any supplement use along with age, sex, ethnicity, BMI, and SES. The analogous models for the cis and trans β-carotene resulted in adjusted OR of 0.7 (95% CI 0.6–0.8; p<0.001). Odds ratios were consistently high and significant for the pollutant factors Heptachlor Epoxide and PCB170 after further analogous adjustment of recent consumption and total lipid levels, with odds ratios of 1.6 (95% CI 1.3–2.1; p<0.001) and 2.2 (95% CI 1.4–3.5; p = 0.003) respectively (Table S4). We concluded that recent consumption as encoded by the dietary recall questionnaire in conjunction with lipid levels did not alter the validity of the associations of the 5 environmental factors found.

To summarize of our secondary tests for validity, we concluded that reverse causality, recent food and supplement consumption, and total lipid levels did not substantially bias our effect estimates for the 5 validated factors. These tests were made possible by the extensive list of co-variates available in the NHANES.

## Discussion

We have described a prototype Environmental-Wide Association Study (EWAS) and applied it to the study of Type 2 Diabetes (T2D), and validated many of our significant findings across independent cohorts and confirmed some of them through the literature. This pilot study is made possible by the examination of multiple cohorts present in the nationally representative NHANES dataset. We have rediscovered factors such as carotenes and PCBs with previously known association with T2D. Unexpectedly, we found higher levels of γ-tocopherol were associated with higher likelihood of T2D, independent of dietary intake. Of the components of Vitamin E, γ-tocopherol is the most abundant form in the US diet [36], and makes up to 50% of the total vitamin E in human muscle and adipose tissue [37], two known insulin-target tissues. As γ-tocopherol has been previously suggested as a preventive agent against colon cancer [38], any potential adverse metabolic effects for this vitamin should be studied closely.

Another novel finding was in the significant association between heptachlor epoxide levels and T2D. Heptachlor is a pesticide; most uses of heptachlor were discontinued in the late 1980s [39]. The main source of heptachlor and its breakdown product, heptachlor epoxide, is from food, but heptachlor epoxide is persistent in the environment and can even be passed in breast milk [40]. While a significant association with T2D has been reported across thirty-thousand pesticide applicators who used the pesticide heptachlor [41], to our knowledge, this broad association between heptachlor epoxide and T2D in the general public, as surveyed by NHANES, is novel.

While this study successfully demonstrates a prototype EWAS for T2D, this methodology can be reconfigured to measure the relationship between environmental factors and other disorders, such as obesity, lipid level abnormalities, hypertension, and/or cardiovascular disease. Methodologically, the EWAS takes inspiration from GWAS, which have been used to assess the correlation between genome-wide variability and disease.

Like GWAS, the utility of EWAS lies in two types of hypothesis generation. First, the EWAS framework can be used to propose targets for further study. For example, many factors are correlated; some are similar structurally, such as the isomers of β-carotene, or co-occur in the environment, such as the PCBs and organochlorine pesticides. As we extend the GWAS analogy, these and other environmental factors could be said to be in “linkage disequilibrium” with each other. Just as is done for preliminary GWAS findings, EWAS findings can and should be used to identify further factors that may be in “disequilibrium,” for further detailed measurement and causal identification.

We acknowledge that the measurement of 266 environmental factors is hardly a comprehensive study of the environment, but this is still a greater number of factors measured than the 30 microsatellite markers [42], or 100 single nucleotide polymorphisms (SNPs) in some of the earliest implementations of GWAS [43]. We suggest that measurement technologies for the environment can and will improve in resolution, as novel associations are made using even few measurements in these prototype studies. Measurement of the panel of environmental factors used here, most of which are performed by mass spectrometry, currently costs an estimated $40,000 per individual [44], or close to the current pricing for whole-genome sequencing.

Another type of hypothesis we may generate is regarding the complex cause of disease. For example, we can now use an EWAS to hypothesize about “gene-environment” interactions and their relation to disease etiology. A future study addressing gene-environment interactions might be designed as a combination of both a GWAS and EWAS, where genetic variability is assessed simultaneously along with key environmental factors. While marginally more resource intensive, this type of study design could perhaps facilitate an explanation of disease causation that has eluded genomic-wide scans in addition and provide more accurate estimates of attributable risk.

The EWAS allows for comprehensive and systematic analysis of the effects of the environment in association with disease on a broad scale. While many investigators have already utilized the NHANES to address the effect of a limited number of factors on disease, they do not provide a global view of these associations [45], [46]. Further, while arriving at similar results, the previous studies use differing definitions of T2D status (medical questionnaire), exposure coding (discretization or log transformation), and lack methods for multiple comparison control [47]–[49]. It is the well-established toolkit of the GWAS that has provided us with methods to overcome these limitations and to enable us to postulate about environment-wide association with disease.

Limitations of this study remind us that measuring environment-wide aspects in relation to phenotypic states such as disease will be a difficult undertaking [50]. Unlike genetic loci, the environment is boundless. While the NHANES provides a large number of factors to study, a comprehensive assessment will require precise definition over a broader dimension (more factors). While laboratory measurements are collected during a baseline fasting state for all participants in NHANES, we will still have to account for the dynamic and heterogeneous nature of different exposures and their associated responses by taking replicate measurements at different physiological states. Further, this study utilizes cross-sectional data and can only show correlation between exposure and disease prevalence. To ascertain causality, we would need to perform prospective EWAS over the life course, consider incident cases, and/or consider randomization methods [51] as additional validation. Due to the number of hypotheses generated, we would need to integrate more evidence from large-scale collaborative studies in order to confirm (or refute) etiological aspects of these factors while being as comprehensive as possible in the observation of potential confounding variables. For example, additional factors such as behavior (food consumption, drug use, and/or exercise patterns), geographic location, and occupation must also be ascertained to account for associated risk factors and reverse causality.

While GWAS has allowed us to find novel variants associated with T2D of possible mechanistic importance and provided a model for a comprehensive study of the environment described here, associated variants have had only moderate effect sizes to date. Most of the risk loci identified with GWAS have small individual odds ratios, generally less than 1.3 [52]–[54] and the highest has been reported to be 1.71, belonging to a variant in the *TCF7L2* gene [55], [56]. Albeit from different populations and analytical scenarios, the effect sizes of our validated environmental factors on T2D were comparable to the highest odds ratios seen in GWAS.

However, the correlated and dynamic nature of a multitude of environmental factors will hinder causal inference to a greater degree than GWAS [50]. Nevertheless, similar biases do influence GWAS interpretation. For example, the statistical association of a variant of *FTO* with T2D was nullified by accounting for BMI [57]. However, despite these hindrances, we view EWAS similarly to GWAS, a step towards learning about a component that plays a large role in complex disease.

It is imperative not only for epidemiologists and geneticists but also physicians and their patients to understand how multiple environmental factors may influence disease in a systematic fashion. Individuals are already demanding information regarding their “body burdens”, or the number and amount of chemicals present in their system, as evidenced by the “Human Toxome Project” [44], [58]. We must learn how all these factors might contribute to disease in context of other common risk factors to inform our health care practitioners and individuals appropriately. We must conduct our analyses in a non-selective fashion.

In conclusion, the EWAS is a promising way to search and consider potential environmental factors as associated with disease or other clinical phenotypes. These results demand a rethinking and restructuring of studies that study disease in the genomics context. The time is ripe to usher in “enviromics” [59], the study of a wide array of environmental factors in relation to health and biology.

## Supporting Information

File S1 The tables in this file describe the baseline demographics of the NHANES cohorts (1999–2000, 2001–2002, 2003–2004, 2005–2006) per T2D status (Fasting Plasma Glucose >125 mg/dL). T2D cases were determined by a clinical threshold of ≥126 mg/dL fasting blood glucose. Unweighted total samples were similar across cohorts. Age and BMI were significantly different between each of groups and the proportion of sex was significantly different in 3 of the 4 cohorts (male referent group). Low, medium, and high estimates of SES were computed by tertile of poverty index. Low SES (lowest tertile of poverty index) is also associated with T2D status in 3 of the 4 cohorts (3rd tertile SES referent group). Ethnicity was not seen to be associated with T2D status (“white” ethnicity referent group). * denotes unweighted number. All other statistics are weighted.
(0.09 MB DOC)

Figure S1 Trans-β-carotene vs. Diabetes Status for 2001–2002, 2003–2004, and 2005–2006 cohorts. Raw exposure data (log-scale) versus T2D Status (Fasting Plasma Glucose >125 mg/dL) for validated environmental factors. Horizontal line represents the weighted median of the group. Cohort plot symbols consistent with Figure 2 (square: 2001–2002; filled bullet: 2003–2004; circle: 2005–2006).
(1.17 MB DOC)

Figure S2 Cis-β-carotene vs. Diabetes Status for 2001–2002, 2003–2004, 2005–2006 cohorts. Raw exposure data (log-scale) versus T2D Status (Fasting Plasma Glucose >125 mg/dL) for validated environmental factors. Horizontal line represents the weighted median of the group. Cohort plot symbols consistent with Figure 2 (square: 2001–2002; filled bullet: 2003–2004; circle: 2005–2006).
(1.01 MB DOC)

Figure S3 γ-tocopherol vs. Diabetes Status for 1999–2000 and 2003–2004 cohorts. Raw exposure data (log-scale) versus T2D Status (Fasting Plasma Glucose >125 mg/dL) for validated environmental factors. Horizontal line represents the weighted median of the group. Cohort plot symbols consistent with Figure 2 (diamond: 1999–2000; filled bullet: 2003–2004).
(0.83 MB DOC)

Figure S4 Heptachlor Epoxide vs. Diabetes Status for 1999–2000 and 2003–2004 cohorts. Raw exposure data (log-scale) versus T2D Status (Fasting Plasma Glucose >125 mg/dL) for validated environmental factors. Horizontal line represents the weighted median of the group. Cohort plot symbols consistent with Figure 2 (diamond: 1999–2000; filled bullet: 2003–2004).
(0.49 MB DOC)

Figure S5 PCB170 vs. Diabetes Status for 1999–2000 and 2003–2004 cohorts. Raw exposure data (log-scale) versus T2D Status (Fasting Plasma Glucose >125 mg/dL) for validated environmental factors. Horizontal line represents the weighted median of the group. Cohort plot symbols consistent with Figure 2 (diamond: 1999–2000; filled bullet: 2003–2004).
(0.50 MB DOC)

Table S1 Percent of significant (p<0.02, FDR between 10 to 30%) environmental factors found by environmental class and cohort in first stage of T2D association. Number found of total per class is also shown in parentheses. * denotes that there were no factors measured for that particular environmental class as of this writing. There were a total of 21 environmental classes explored.
(0.05 MB DOC)

Table S2 Adjusted odds ratios for validated factors for individuals at risk for T2D diagnosis. Individuals who answered yes to having T2D in the NHANES questionnaire (“Doctor told you have diabetes?”) were omitted from the sample, leaving only those who were at risk for T2D diagnosis. Estimates were adjusted for age, sex, BMI, SES, ethnicity, and cohort. Odds ratios are for a change in 1SD of the logarithm of exposure in association with T2D diagnosis risk.
(0.03 MB DOC)

Table S3 Adjusted odds ratios for validated factors, adjusting for age, sex, BMI, SES, ethnicity, cohort, log10(total triglycerides), log10(total cholesterol). Odds ratios are for a change in 1SD of the logarithm of exposure in association with T2D.
(0.03 MB DOC)

Table S4 Adjusted odds ratios for validated factors, adjusting for age, sex, BMI, SES, ethnicity, cohort, log10(triglycerides), log10(total cholesterol), log10(total vitamin E consumption recall), log10(total β-carotene consumption recall), log10(total carbohydrate consumption recall), supplement use (yes/no) . Odds ratios are for a change in 1 SD of the logarithm of exposure in association with T2D. * denotes models did not include β-carotene as these data were not available for the 1999–2000 cohort.
(0.03 MB DOC)
