# Supplementary material for: An Environment-Wide Association Study (EWAS) on Type 2 Diabetes Mellitus
Source: PLoS One. 2010 May 20;5(5):e10746. doi: 10.1371/journal.pone.0010746 (PMC2873978; doi:10.1371/journal.pone.0010746)
Supplement: File S1 — The tables in this file describe the baseline demographics of the NHANES cohorts (1999–2000, 2001–2002, 2003–2004, 2005–2006) per T2D status (Fasting Plasma Glucose >125 mg/dL). T2D cases were determined by a clinical threshold of ≥126 mg/dL fasting blood glucose. Unweighted total samples were similar across cohorts. Age and BMI were significantly different between each of groups and the proportion of sex was significantly different in 3 of the 4 cohorts (male referent group). Low, medium, and high estimates of SES were computed by tertile of poverty index. Low SES (lowest tertile of poverty index) is also associated with T2D status in 3 of the 4 cohorts (3rd tertile SES referent group). Ethnicity was not seen to be associated with T2D status (“white” ethnicity referent group). * denotes unweighted number. All other statistics are weighted. (0.09 MB DOC) [file pone.0010746.s001.doc]

An Environment-Wide Association Study (EWAS) to Type 2 Diabetes (T2D)

Chirag J Patel, Jayanta Bhattacharya, Atul J Butte

**File S1**

The tables in this file describe the baseline demographics of the NHANES cohorts (1999-2000, 2001-2002, 2003-2004, 2005-2006) per T2D status (Fasting Plasma Glucose > 125 mg/dL).

T2D cases were determined by a clinical threshold of ≥ 126 mg/dL fasting blood glucose. Unweighted total samples were similar across cohorts. Age and BMI were significantly different between each of groups and the proportion of sex was significantly different in 3 of the 4 cohorts (male referent group). Low, medium, and high estimates of SES were computed by tertile of poverty index. Low SES (lowest tertile of poverty index) is also associated with T2D status in 3 of the 4 cohorts (3^rd^ tertile SES referent group). Ethnicity was not seen to be associated with T2D status (“white” ethnicity referent group). * denotes unweighted number. All other statistics are weighted.

**Baseline characteristics for cases (T2D) and controls (no T2D) for NHANES cohort 1999-2000.**

| **1999-2000** | **T2D (N*=197)** | **No T2D (N*=3070)** | **P** |
| --- | --- | --- | --- |
| **Sex** |  |  |  |
| Female N* (%) | 97 (44) | 1596 (52) | 0.03 |
| **Age** |  |  |  |
| Mean (CI) | 54.4 (49.3, 59.5) | 39.8 (38.5, 41.1) | < 0.001 |
| **SES** |  |  |  |
| low N* (%) | 86 (32) | 1084 (27) | 0.03 |
| middle N* (%) | 53 (44) | 856 (33) | 0.03 |
| high N* (%) | 30 (24) | 682 (39) |  |
| **BMI** |  |  |  |
| Mean (CI) | 31.4 (29.5, 33.4) | 26.8 (26.3, 27.3) | < 0.001 |
| **Ethnicity** |  |  |  |
| Black N* (%) | 46 (12) | 666 (10) | 0.4 |
| Mexican-American N* (%) | 61 (6) | 1008 (7) | 0.9 |
| Other Hispanic N* (%) | 16 (11) | 181 (8) | 0.5 |
| Other N* (%) | 7 (4) | 90 (4) | 0.96 |
| White N* (%) | 67 (67) | 1125 (69) |  |

**Baseline characteristics for cases (T2D) and controls (no T2D) for NHANES 2001-2002 cohort**

| **2001-2002** | **T2D (N*=251)** | **No T2D (N*=3415)** | **P** |
| --- | --- | --- | --- |
| **Sex** |  |  |  |
| Female N* (%) | 111 (35) | 1785 (53) | < 0.001 |
| **Age** |  |  |  |
| Mean (CI) | 56.8 (54.1, 59.5) | 40.1 (38.4, 41.7) | < 0.001 |
| **SES** |  |  |  |
| low N* (%) | 95 (36) | 1271 (30) | 0.03 |
| middle N* (%) | 84 (38) | 1064 (34) | 0.22 |
| high N* (%) | 52 (26) | 843 (36) |  |
| **BMI** |  |  |  |
| Mean (CI) | 32.2 (30.2, 34.3) | 27.0 (26.8, 27.2) | < 0.001 |
| **Ethnicity** |  |  |  |
| Black N* (%) | 51 (12) | 795 (11) | 0.4 |
| Mexican-American N* (%) | 62 (7) | 820 (7) | 0.9 |
| Other Hispanic N* (%) | 12 (6) | 123 (5) | 0.3 |
| Other N* (%) | 10 (9) | 128 (5) | 0.3 |
| White N* (%) | 116 (65) | 1549 (71) |  |

**Baseline characteristics for cases (T2D) and controls (no T2D) for NHANES 2003-2004 cohort.**

| **2003-2004** | **T2D (N*=228)** | **No T2D (N*=3128)** | **P** |
| --- | --- | --- | --- |
| **Sex** |  |  |  |
| Female N* (%) | 103 (39) | 1592 (52) | 0.02 |
| **Age** |  |  |  |
| Mean (CI) | 57.7 (54.2, 61.2) | 40.9 (39.5, 42.3) | < 0.001 |
| **SES** |  |  |  |
| low N* (%) | 89 (28) | 1330 (31) | 0.17 |
| middle N* (%) | 74 (40) | 877 (32) | 0.12 |
| high N* (%) | 51 (32) | 738 (37) |  |
| **BMI** |  |  |  |
| Mean (CI) | 31.9 (30.6,33.3) | 27.5 (27.2, 27.8) | < 0.001 |
| **Ethnicity** |  |  |  |
| Black N* (%) | 52 (14) | 816 (12) | 0.2 |
| Mexican-American N* (%) | 70 (12) | 700 (8) | 0.3 |
| Other Hispanic N* (%) | 8 (7) | 90 (3) | 0.3 |
| Other N* (%) | 7 (7) | 133 (6) | 0.4 |
| White N* (%) | 91 (60) | 1389 (71) |  |

**Baseline characteristics for cases (T2D) and controls (no T2D) for NHANES cohort 2005-2006.**

| **2005-2006** | **T2D (N*=234)** | **No T2D (N*=3118)** | **P** |
| --- | --- | --- | --- |
| **Sex** |  |  |  |
| Female N* (%) | 119 (56) | 1575 (52) | 0.5 |
| **Age** |  |  |  |
| Mean (CI) | 58.5 (56, 61.1) | 41.0 (39.4, 42.7) | < 0.001 |
| **SES** |  |  |  |
| low N* (%) | 114 (37) | 1345 (30) | 0.006 |
| middle N* (%) | 66 (41) | 917 (35) | 0.03 |
| high N* (%) | 33 (21) | 712 (36) |  |
| **BMI** |  |  |  |
| Mean (CI) | 33.3 (32.2, 34.5) | 27.7 (27.2, 28.2) | < 0.001 |
| **Ethnicity** |  |  |  |
| Black N* (%) | 61 (12) | 830 (12) | 0.6 |
| Mexican-American N* (%) | 59 (11) | 737 (8) | 0.2 |
| Other Hispanic N* (%) | 5 (5) | 101 (3) | 0.6 |
| Other N* (%) | 5 (4) | 142 (6) | 0.4 |
| White N* (%) | 104 (68) | 1308 (71) |  |
