# Supplementary material for: An Environment-Wide Association Study (EWAS) on Type 2 Diabetes Mellitus
Source: PLoS One. 2010 May 20;5(5):e10746. doi: 10.1371/journal.pone.0010746 (PMC2873978; doi:10.1371/journal.pone.0010746)

An Environment-Wide Association Study (EWAS) to Type 2 Diabetes (T2D)

Chirag J Patel, Jayanta Bhattacharya, Atul J Butte

**Figure S2:**

Cis-β-carotene vs. Diabetes Status for 2001-2002, 2003-2004, 2005-2006 cohorts. Raw exposure data (log-scale) versus T2D Status (Fasting Plasma Glucose > 125 mg/dL) for validated environmental factors. Horizontal line represents the weighted median of the group. Cohort plot symbols consistent with Figure 2 (square: 2001-2002; filled bullet: 2003-2004; circle: 2005-2006).


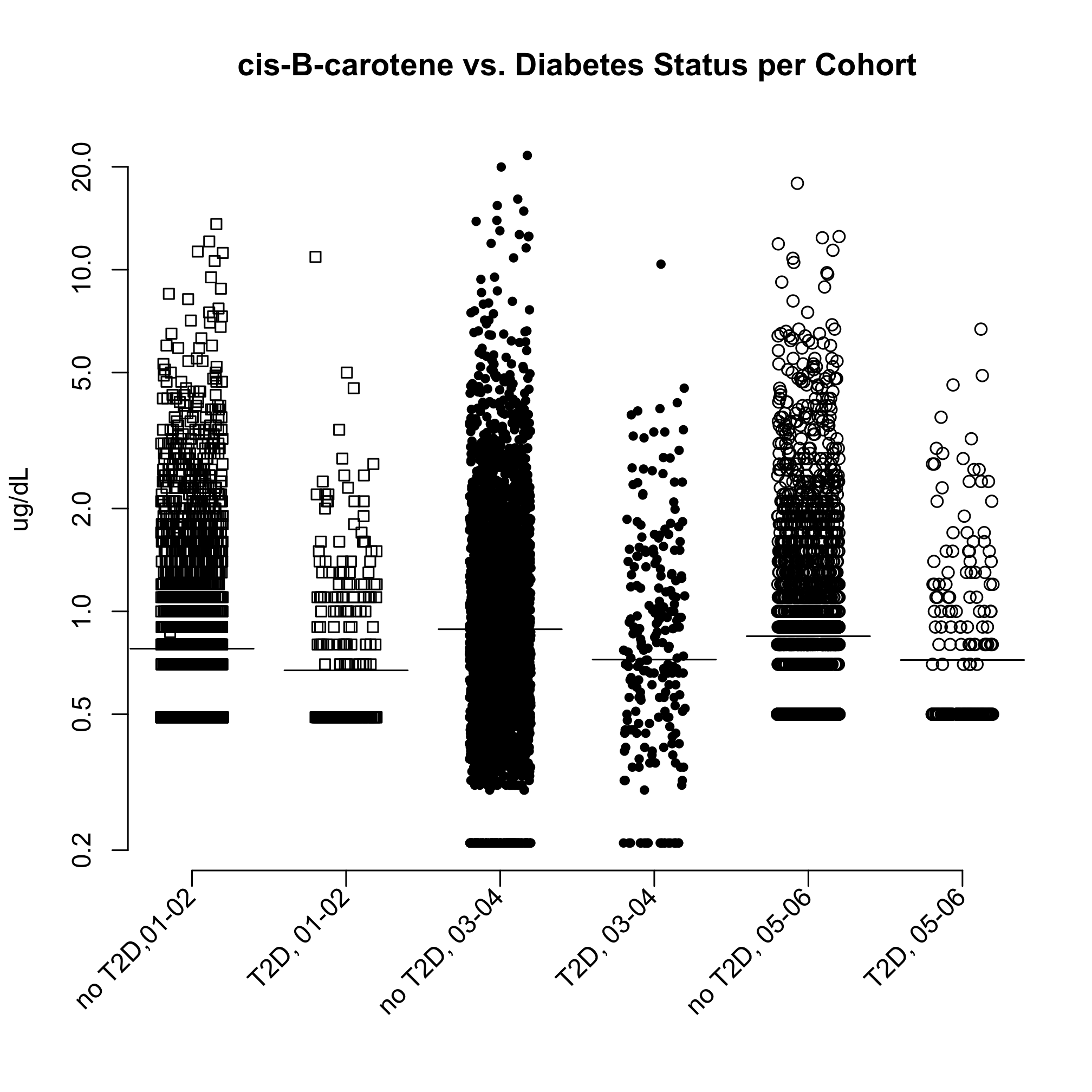

Supplement: Figure S2 — Cis-β-carotene vs. Diabetes Status for 2001–2002, 2003–2004, 2005–2006 cohorts. Raw exposure data (log-scale) versus T2D Status (Fasting Plasma Glucose >125 mg/dL) for validated environmental factors. Horizontal line represents the weighted median of the group. Cohort plot symbols consistent with Figure 2 (square: 2001–2002; filled bullet: 2003–2004; circle: 2005–2006). (1.01 MB DOC) [file pone.0010746.s003.doc]
