# Supplementary material for: An Environment-Wide Association Study (EWAS) on Type 2 Diabetes Mellitus
Source: PLoS One. 2010 May 20;5(5):e10746. doi: 10.1371/journal.pone.0010746 (PMC2873978; doi:10.1371/journal.pone.0010746)

An Environment-Wide Association Study (EWAS) to Type 2 Diabetes (T2D)

Chirag J Patel, Jayanta Bhattacharya, Atul J Butte

**Figure S4:**

Heptachlor Epoxide vs. Diabetes Status for 1999-2000 and 2003-2004 cohorts. Raw exposure data (log-scale) versus T2D Status (Fasting Plasma Glucose > 125 mg/dL) for validated environmental factors. Horizontal line represents the weighted median of the group. Cohort plot symbols consistent with Figure 2 (diamond: 1999-2000; filled bullet: 2003-2004).


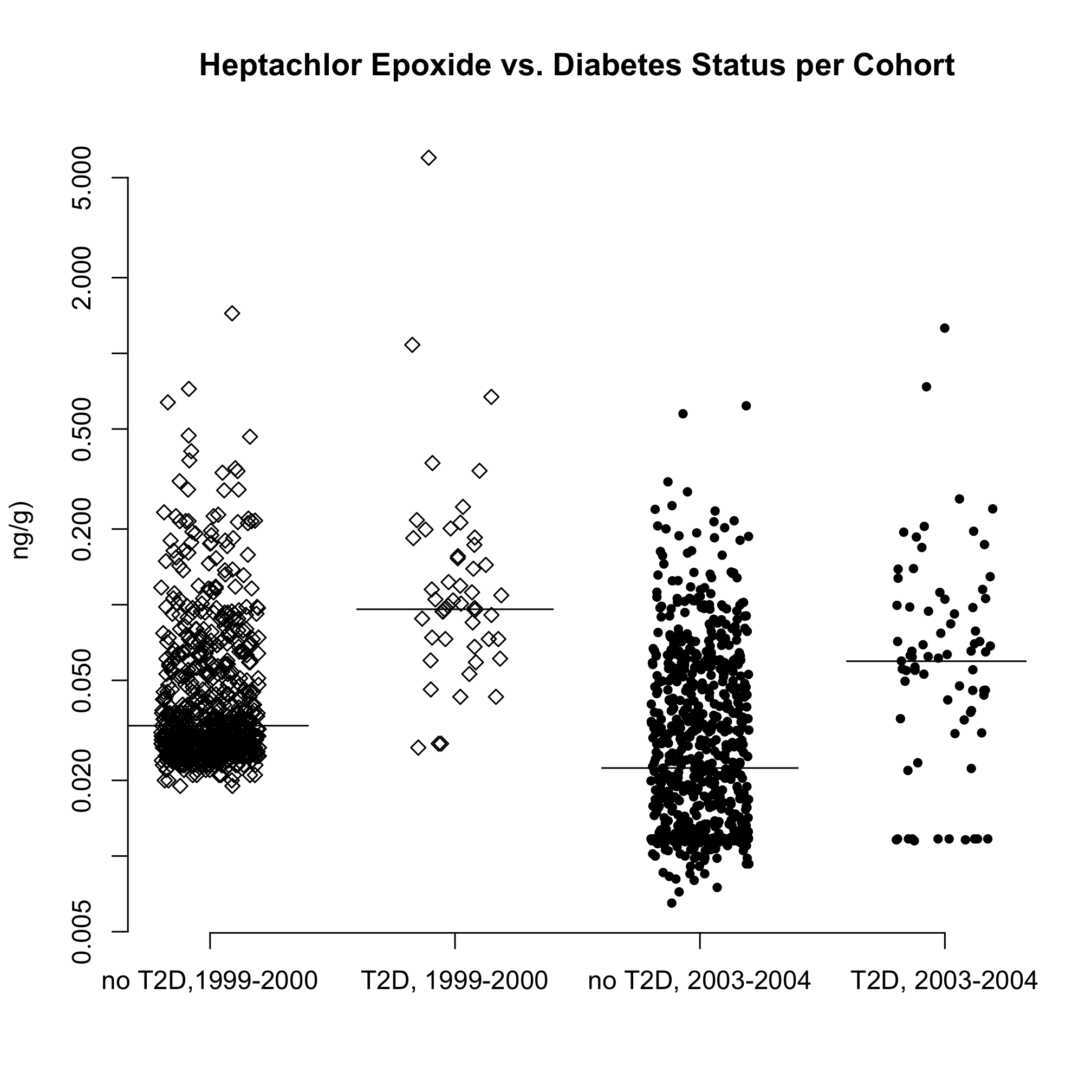

Supplement: Figure S4 — Heptachlor Epoxide vs. Diabetes Status for 1999–2000 and 2003–2004 cohorts. Raw exposure data (log-scale) versus T2D Status (Fasting Plasma Glucose >125 mg/dL) for validated environmental factors. Horizontal line represents the weighted median of the group. Cohort plot symbols consistent with Figure 2 (diamond: 1999–2000; filled bullet: 2003–2004). (0.49 MB DOC) [file pone.0010746.s005.doc]
