# Supplementary material for: An Environment-Wide Association Study (EWAS) on Type 2 Diabetes Mellitus
Source: PLoS One. 2010 May 20;5(5):e10746. doi: 10.1371/journal.pone.0010746 (PMC2873978; doi:10.1371/journal.pone.0010746)
Supplement: Table S1 — Percent of significant (p<0.02, FDR between 10 to 30%) environmental factors found by environmental class and cohort in first stage of T2D association. Number found of total per class is also shown in parentheses. * denotes that there were no factors measured for that particular environmental class as of this writing. There were a total of 21 environmental classes explored. (0.05 MB DOC) [file pone.0010746.s007.doc]

An Environment-Wide Association Study (EWAS) to Type 2 Diabetes (T2D)

Chirag J Patel, Jayanta Bhattacharya, Atul J Butte

***Table S1****.* **Percent of significant (p < 0.02, FDR between 10 to 30%) environmental factors found by environmental class and cohort in first stage of T2D association**.

|  | **1999-2000** | **2001-2002** | **2003-2004** | **2005-2006** |
| --- | --- | --- | --- | --- |
| nutrients & vitamins | 8 (1 of 13) | 21 (4 of 19) | 16 (5 of 32) | 17 (3 of 18) |
| organochlorine pesticides | 29 (2 of 7) | 0 (0 of 9) | 10 (1 of 10) | * |
| polychlorinated biphenyls | 15 (2 of 13) | 0 (0 of 20) | 29 (11 of 38) | * |
| heavy metals | 0 (0 of 15) | 0 (0 of 16) | 10 (2 of 20) | 5 (1 of 21) |
| virus | 25 (1 of 4) | 0 (0 of 4) | 25 (1 of 4) | 0 (0 of 4) |
| dioxins | 0 (0 of 5) | 0 (0 of 6) | 29 (2 of 7) | * |
| furans | 0 (0 of 4) | 0 (0 of 4) | 20 (1 of 5) | * |
| volatile compounds | * | 0 (0 of 10) | 0 (0 of 21) | * |
| other pesticides | 0 (0 of 3) | 0 (0 of 1) | 0 (0 of 4) | 0 (0 of 1) |
| phenols | 0 (0 of 11) | 0 (0 of 8) | 0 (0 of 9) | 0 ( 0 of 11) |
| phthalates | 0 (0 of 13) | 0 (0 of 17) | 0 (0 of 16) | * |
| allergen tests | * | * | * | 0 (0 of 11) |
| bacteria | 0 (0 of 3) | 0 (0 of 3) | 0 (0 of 3) | * |
| cotinine | 0 (0 of 1) | 0 ( 0 of 1) | 0 (0 of 1) | 0 (0 of 1) |
| diakyls | 0 (0 of 7) | 0 (0 of 7) | 0 (0 of 6) | * |
| hydrocarbons | 0 (0 of 11) | 0 ( 0 of 21) | 0 (0 of 13) | * |
| latex | 0 (0 of 1) | * | * | * |
| perchlorate | * | * | 0 (0 of 2) | * |
| polybrominated ethers | * | * | 0 (0 of 11) | * |
| polyflourochemicals | * | * | 0 (0 of 7) | 0 (0 of 8) |
| acrylamide | * | * | 0 (0 of 2) | * |

Number found of total per class is also shown in parentheses. * denotes that there were no factors measured for that particular environmental class as of this writing. There were a total of 21 environmental classes explored.
