# Supplementary material for: An Environment-Wide Association Study (EWAS) on Type 2 Diabetes Mellitus
Source: PLoS One. 2010 May 20;5(5):e10746. doi: 10.1371/journal.pone.0010746 (PMC2873978; doi:10.1371/journal.pone.0010746)
Supplement: Table S2 — Adjusted odds ratios for validated factors for individuals at risk for T2D diagnosis. Individuals who answered yes to having T2D in the NHANES questionnaire (“Doctor told you have diabetes?”) were omitted from the sample, leaving only those who were at risk for T2D diagnosis. Estimates were adjusted for age, sex, BMI, SES, ethnicity, and cohort. Odds ratios are for a change in 1SD of the logarithm of exposure in association with T2D diagnosis risk. (0.03 MB DOC) [file pone.0010746.s008.doc]

An Environment-Wide Association Study (EWAS) to Type 2 Diabetes (T2D)

Chirag J Patel, Jayanta Bhattacharya, Atul J Butte

***Table S2.*** **Adjusted odds ratios for validated factors for individuals at risk for T2D diagnosis.**

| **Environmental class** | **Environment Factor** | **Cohort** | **P** | **OR**  **(95% CI)** |
| --- | --- | --- | --- | --- |
| Nutrients | cis-β-carotene | 2001-2006 | < 0.001 | 0.6 (0.5-0.7) |
|  | trans-β-carotene | 2001-2006 | 0.001 | 0.7 (0.5-0.8) |
|  | γ-tocopherol | 1999-2006 | < 0.001 | 1.8 (1.3-2.2) |
| Organochlorine Pesticides | Heptachlor Epoxide | 1999-2004 | 0.008 | 1.6 (1.1-2.1) |
| Polychlorinated Biphenyls | PCB170 | 1999-2004 | 0.02 | 2.1 (1.2-3.9) |

Individuals who answered yes to having T2D in the NHANES questionnaire (“Doctor told you have diabetes?”) were omitted from the sample, leaving only those who were at risk for T2D diagnosis. Estimates were adjusted for age, sex, BMI, SES, ethnicity, and cohort. Odds ratios are for a change in 1SD of the logarithm of exposure in association with T2D diagnosis risk.
