# Supplementary material for: An Environment-Wide Association Study (EWAS) on Type 2 Diabetes Mellitus
Source: PLoS One. 2010 May 20;5(5):e10746. doi: 10.1371/journal.pone.0010746 (PMC2873978; doi:10.1371/journal.pone.0010746)
Supplement: Table S4 — Adjusted odds ratios for validated factors, adjusting for age, sex, BMI, SES, ethnicity, cohort, log10(triglycerides), log10(total cholesterol), log10(total vitamin E consumption recall), log10(total β-carotene consumption recall), log10(total carbohydrate consumption recall), supplement use (yes/no) . Odds ratios are for a change in 1 SD of the logarithm of exposure in association with T2D. * denotes models did not include β-carotene as these data were not available for the 1999–2000 cohort. (0.03 MB DOC) [file pone.0010746.s010.doc]

An Environment-Wide Association Study (EWAS) to Type 2 Diabetes (T2D)

Chirag J Patel, Jayanta Bhattacharya, Atul J Butte

***Table S4***. **Adjusted odds ratios for validated factors, adjusting for age, sex, BMI, SES, ethnicity, cohort, log10(triglycerides), log10(total cholesterol), log10(total vitamin E consumption recall), log10(total β-carotene consumption recall), log10(total carbohydrate consumption recall), supplement use (yes/no) .**

| **Environmental class** | **Environment Factor** | **Cohort** | **P** | **OR**  **(95% CI)** |
| --- | --- | --- | --- | --- |
| Nutrients | cis-β-carotene | 2001-2006 | < 0.001 | 0.7 (0.6, 0.8) |
|  | trans-β-carotene | 2001-2006 | < 0.001 | 0.7 (0.6, 0.8) |
|  | γ-tocopherol | 1999-2006 | 0.006 | 1.3 (1.1, 1.5) |
| Organochlorine Pesticides | Heptachlor Epoxide | 1999-2004 | < 0.001 | 1.6* (1.3, 2.1) |
| Polychlorinated Biphenyls | PCB170 | 1999-2004 | 0.003 | 2.2* (1.4, 3.5) |

Odds ratios are for a change in 1 SD of the logarithm of exposure in association with T2D. * denotes models did not include β-carotene as these data were not available for the 1999-2000 cohort.
